# Chestnut resistance to the blight disease: insights from transcriptome analysis

**DOI:** 10.1186/1471-2229-12-38

**Published:** 2012-03-19

**Authors:** Abdelali Barakat, Meg Staton, Chun-Huai Cheng, Joseph Park, Norzawani Buang M Yassin, Stephen Ficklin, Chia-Chun Yeh, Fred Hebard, Kathleen Baier, William Powell, Stephan C Schuster, Nicholas Wheeler, Albert Abbott, John E Carlson, Ronald Sederoff

**Affiliations:** 1 The School of Forest Resources, and The Huck Institutes of the Life Sciences, Pennsylvania State University, 326 Forest Resources Building, University Park, PA 16802, USA; 2 Clemson University Genomics Institute, Clemson University, 310 Biosystems Research Complex, 51 New Cherry Street,, Clemson, SC 29631, USA; 3 Meadowview Research Farms, Meadowview, VA 24361-3349, USA; 4 Department Biochemistry and Molecular Biology, Pennsylvania State University, 310 Wartik laboratory, University Park, PA 16802, USA; 5 Department of Forestry and Environmental Resources, North Carolina State University, Campus Box, 7247, Raleigh, NC 27695, USA; 6 Department of Biochemistry and Genetics, Clemson University, 116 Jordan Hall, Clemson, SC 29631, USA; 7 College of Environmental Science & Forestry, State University of New York, One Forestry Drive, Syracuse, NY 13210-2788, USA; 8 Department of Bioenergy Science and Technology, Chonnam National University, Buk-Gu, Gwangju 500-757, Korea

## Abstract

**Background:**

A century ago, Chestnut Blight Disease (CBD) devastated the American chestnut. Backcross breeding has been underway to introgress resistance from Chinese chestnut into surviving American chestnut genotypes. Development of genomic resources for the family Fagaceae, has focused in this project on *Castanea mollissima *Blume (Chinese chestnut) and *Castanea dentata *(Marsh.) Borkh *(*American chestnut*) *to aid in the backcross breeding effort and in the eventual identification of blight resistance genes through genomic sequencing and map based cloning. A previous study reported partial characterization of the transcriptomes from these two species. Here, further analyses of a larger dataset and assemblies including both 454 and capillary sequences were performed and defense related genes with differential transcript abundance (GDTA) in canker versus healthy stem tissues were identified.

**Results:**

Over one and a half million cDNA reads were assembled into 34,800 transcript contigs from American chestnut and 48,335 transcript contigs from Chinese chestnut. Chestnut cDNA showed higher coding sequence similarity to genes in other woody plants than in herbaceous species. The number of genes tagged, the length of coding sequences, and the numbers of tagged members within gene families showed that the cDNA dataset provides a good resource for studying the American and Chinese chestnut transcriptomes. *In silico *analysis of transcript abundance identified hundreds of GDTA in canker versus healthy stem tissues. A significant number of additional DTA genes involved in the defense-response not reported in a previous study were identified here. These DTA genes belong to various pathways involving cell wall biosynthesis, reactive oxygen species (ROS), salicylic acid (SA), ethylene, jasmonic acid (JA), abscissic acid (ABA), and hormone signalling. DTA genes were also identified in the hypersensitive response and programmed cell death (PCD) pathways. These DTA genes are candidates for host resistance to the chestnut blight fungus, *Cryphonectria parasitica*.

**Conclusions:**

Our data allowed the identification of many genes and gene network candidates for host resistance to the chestnut blight fungus, *Cryphonectria parasitica*. The similar set of GDTAs in American chestnut and Chinese chestnut suggests that the variation in sensitivity to this pathogen between these species may be the result of different timing and amplitude of the response of the two to the pathogen infection. Resources developed in this study are useful for functional genomics, comparative genomics, resistance breeding and phylogenetics in the Fagaceae.

## Background

The family Fagaceae includes more than 900 species in nine genera [1] found in temperate, subtropical, and tropical regions of the world [2]. In the Western Hemisphere, the Fagaceae range from southern Canada to Colombia [1] where they grow as tall trees or, occasionally, as shrubs. The Fagaceae include major species such as chestnuts, oaks, and beeches [2]. The Fagaceae have significant ecological and economic value. They are predominant species of most hardwood forests in the Northern Hemisphere. Nuts of *Fagus *(beeches)*, Castanea *(chestnuts), *Quercus *(oaks) and most *Castanopsis *species are important sources of food for forest animals and produce a high quality extractable oil [3]. The Fagaceae are important as a timber resource for construction, telephone poles, floors, furniture, cabinets, and other applications. American chestnut played an especially important economic and ecological role by providing food to various insects, birds, and mammals, and food, fiber, and wood for rural communities, prior to the introduction of the Chestnut Blight Disease (CBD) early in the 20th century [4].

**Figure 1:**
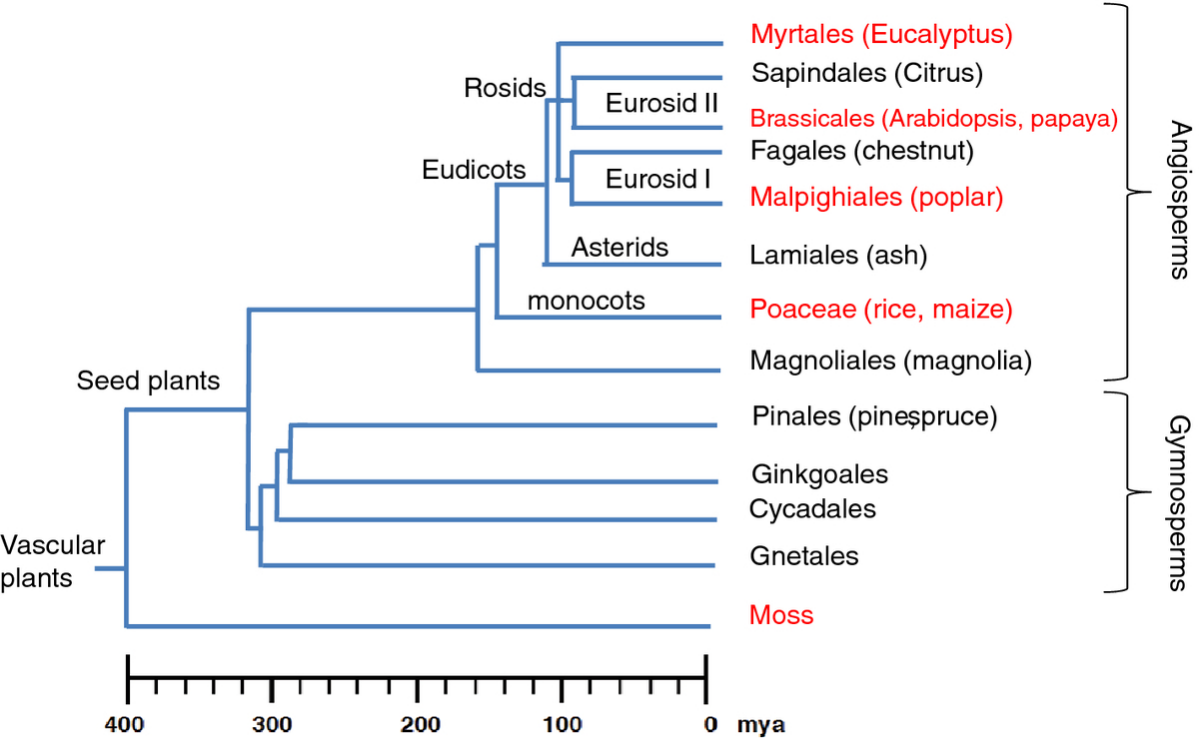
**Simplified phylogeny of the major groups of woody plants**. Species for which genome sequencing has been completed are indicated in red. (Modified from [7].

Fossil dating reveals that species of the order Fagales existed 84 million years ago (Mya) [5]. The time of divergence of the families of the Fagales has been estimated to be as early as 103 mya [6]. Fagaceae species hold a key position in the phylogeny of angiosperms (Figure 1); they cluster closely to Cucurbitales in the Eurosid I group, which includes several model plant species, such as *Medicago truncatula*, *Glycine max*, *Populus trichocarpa*, and *Eucalyptus grandis *for which complete or nearly complete whole-genome sequences exist. The close phylogenetic relationships of several model plants should make members of the Fagaceae good models for comparative genomics between woody and herbaceous species.

Genomic resources for the Fagaceae also have practical applications. CBD is a stem canker disease caused by the fungus *Cryphonectria parasitica*. The pathogen infects stem tissues and kills the above ground portions of trees by girdling the cambium. Today, American chestnut exists primarily as an understory shrub, repeatedly sprouting from the root collar of blight-topped trees [8,9]. Programs to breed timber-type blight resistant chestnut were initiated after appearance of the blight [8], but failed to produce the desired timber-type tree with acceptable levels of resistance. In 1983, a program was initiated by the American Chestnut Foundation [10] to introgress genes from blight-resistant Asian chestnut species into American chestnut via backcross breeding [11]. The American Chestnut Cooperators Foundation, has made crosses between pure American chestnut trees that have limited resistance [8] for future selection. Introductions of hypovirulent fungal genotypes of *C*. *parasitica *[12], have successfully controlled the severity of blight in Europe, but have had only limited success in North America. Understanding the molecular basis of resistance to the CBD could enable the eventual restoration of American chestnut to the eastern North American forests [13].

The Genomic Tool Development for the Fagaceae (GTDF) project [14] was organized to develop genomic resources for several North American Fagaceae species to help address major forest health problems of American chestnut and other trees within this botanical family. The over 1.5 million cDNA sequences generated in this study for American and Chinese chestnut have been used to advance knowledge about the genetics of resistance to the CBD. A preliminary comparison of transcriptome sequences of healthy and blight infected American chestnut and Chinese chestnut resulted in the identification of potential candidate disease resistance genes [15]. However, the 2009 report was based on only 13,000 and 15,000 contigs for American chestnut and Chinese chestnut, respectively, available at the time. We now report on over 83,000 contigs in total for the two species, which includes 9000 Sanger sequences from Chinese chestnut in addition to 454 sequence reads. Moreover, the assembly of transcript contigs was performed using the SeqMan™ NGen™ v1.2 software (DNAStar, Inc) which provided better assembly of the 454 reads than the version of the Newbler program available in 2008. The orthologs used for comparison of DTA genes between American and Chinese chestnut were better defined in this study through the use of a reciprocal best hits analysis. The DEGseq software package [16] used for assessing transcript abundance (TA) includes statistical analyses that make it a more powerful tool than the one used the previous analysis.

This paper reports on further analysis of the transcriptome of chestnut and the identification of candidate genes involved in the defense against CBD. The coverage of the transcriptome was analyzed for number of genes identified, sequence length, gene family size, and similarity to the transcriptome of other woody and herbaceous species. The transcript contigs include full length coding sequences from American and Chinese chestnut respectively which represents a valuable resource for genomic studies in the Fagaceae. The analysis of the differential transcript abundance (DTA) of chestnut genes using DEGseq [16] allowed the identification of hundreds of defense related candidate genes with DTA in canker versus healthy stem tissues. A small set of candidate genes for resistance to CBD were verified for their DTA using real time quantitative RT-PCR.

## Results

### Sequencing summary

More than 1.5 million (1,526,670) reads were generated corresponding to 400 million nucleotides of cDNA sequences from the two studied species. Transcript contigs were assembled from the pyrosequencing reads using Newbler software (Roche 454) and designated version 1 on the Fagaceae website [14]. A second assembly, performed on sequences generated from both pyrosequencing and capillary sequencing reads using SeqMan™ NGen™ v1.2 software (DNAStar, Inc), was designated version 2. The version 2 contig set included longer contigs and more sequences were integrated into the contigs when compared to the original 454 Newbler assemblies. The combination of Sanger sequences and 454 sequences also resulted in slightly fewer but longer contigs. General information about the sequences and contigs identified from each species are summarized (Tables 1 and 2).

**Table 1:** Sources of tissues and 454 sequencing results per tissue for the American and Chinese chestnut EST Datasets.

Library	Tissues Sampled	Genotype	# 454 plates	Ave. ^1 ^Read Length, bp	Total # Reads	Total bp Sequence
ACCanker	American chestnut cankers (infected stems)	TACF^2^, BA69	1	101	129,508	13,080,308

ACHS1	American chestnut healthy stems	CAES^3^, Wisneiwski	3/4	247	222,939	29,828,910

ACHS2	American chestnut healthy stems	CAES, Watertown	3/4	246	254,810	38,165,054

ACWP1	American chestnut whole plant tissues, pooled	CAES, Wisneiwski	1/4	239	47,653	11,380,607

ACWP2	American chestnut whole plant tissues, pooled	CAES, Watertown	1/4	221	33,288	7,364,484

AC Totals	5 cDNA libraries	3 genotypes	3	210.8	688,198	99,819,363

CCCanker	Chinese chestnut var Nanking, cankers	TACF, VA37	1	101	235,635	23,799,135

CCMHS	Chinese chestnut var Mahogany, healthy stems	TACF, BX316	3/4	246	228,594	56,051,191

CCNHS	ChinesecChestnut var Nanking, healthy stems	TACF, GR119	3/4	247	259,859	64,271,926

CCWP1	Chinese chestnut var Nanking, whole plant tissues	TACF, GR119,	1/4	242	60,445	14,643,040

CCWP2	Chinese chestnut var Mahogany whole plant tissues	TACF, BX316;	1/4	246	53,939	13,249,397

CC Total	5 cDNA libraries	3 genotypes	3	205	838,472	172,014,689

^1^, Ave, average; ^2^, TACF, The American Chestnut Foundation's Meadowview VA Farm; ^3^, CAES, Connecticut Agricultural Research Station. (NB: Canker tissue RNAs were sequenced with the GS20 while other RNAs were sequenced using the FLX version of the 454 system)

**Table 2:** Results of mass assembly of sequence reads from all libraries for each of American chestnut and Chinese chestnut into contigs^1^.

Reads				Assembly V1				Assembly V2		
**Species**	**# 454 plates**	**# 454 reads**	**# of bp**	**AL^2 ^of reads**	**#contigs**	**AL. of contigs**	**% of reads in contigs**	**# contigs**	**AL of contigs**	**% reads in contigs**

ACa^3^	2	398,783	78,183,046	197 bp	22,714	374	59.4%	NA	NA	NA

ACb^4^	1	688,198	146,479,551	200	NA	NA	NA	34,800	412	80.9%

CCa^5^	3	838,472	171,849,098	207	32,738	501	66.8%	48,501	526	87.4%

CCb^6^	3	838,472	171,849,098	207	NA	NA	NA	48,335	543	87.7%

^1^, All of the assemblies are available for download at the http://www.fagaceae.org.website; ^2^, AL, Average length; ^3^, American chestnut, 2 initial 454 runs; ^4^, ACb, American chestnut, 3 454 runs; ^5^, CCa, Chinese chestnut 454 reads only; ^6^, CCb, Chinese chestnut 454 and Sanger reads combined; ^7^, NA, Not appropriate. (NB: Version 1 assemblies were conducted 1-31-2008 with 454 Newbler software; Version 2 assemblies were conducted 8-15-2008 with SeqManPro software)

### Analysis of the American and Chinese chestnut transcriptomes

**Figure 2:**
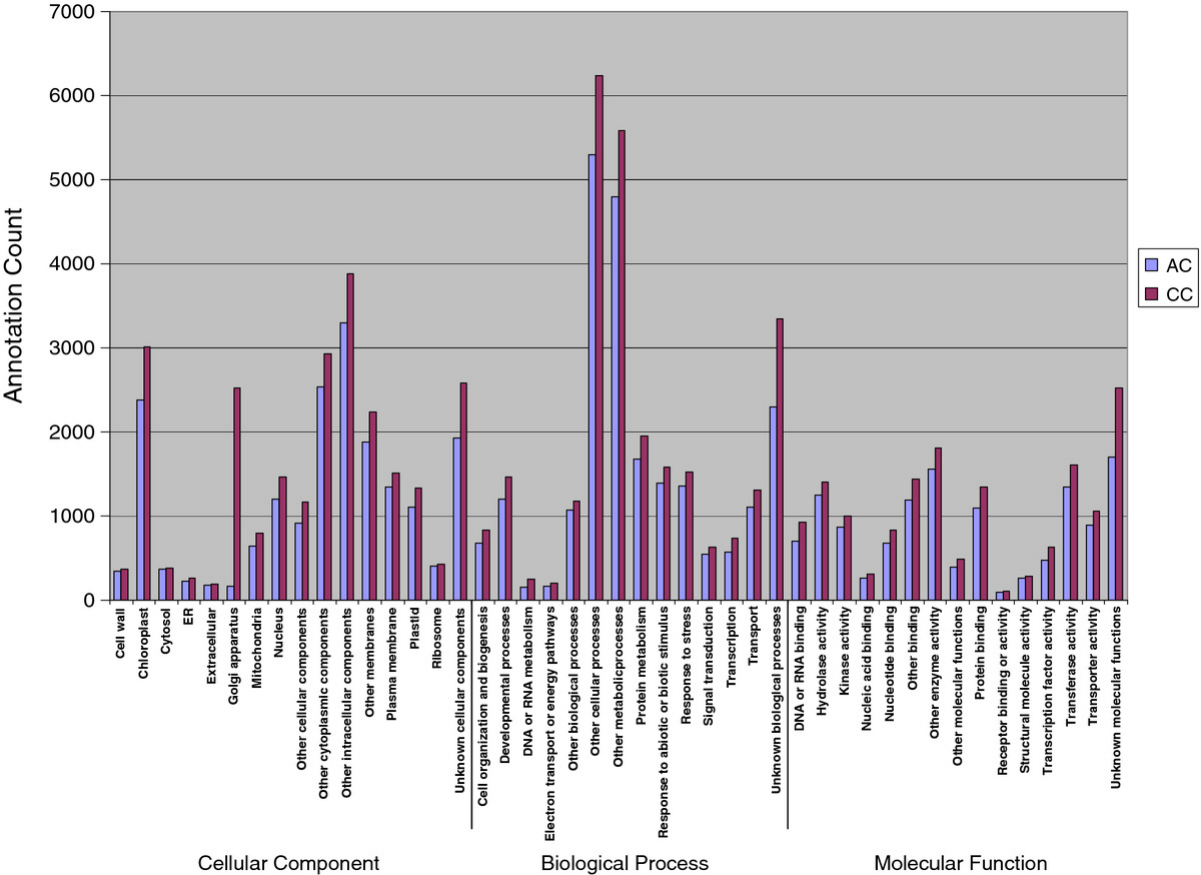
**Histogram presentation of Gene Ontology classification of putative biological processes of contigs from American chestnut (AC), and Chinese chestnut (CC)**. The Y axis indicates the annotation count corresponding to each biological process indicated on the X axis.

For the two Fagaceae species in this study, over one and half million sequencing reads were generated and yielded a total of 93,018 contigs in total for the separate assemblies of the cDNA libraries for the 10 tissues sampled. A small fraction of contigs matched mitochondrial (1.3%) and chloroplast (3%) genes. Similarly, ~2.5% of American and Chinese chestnut sequences obtained from canker tissues had best BLASTX alignments to the *Cryphonectria parasitica *proteome. Transcriptome assembly, version 2 (using all of the reads combined across all tissues), led to the identification of 34,800 and 48,501 contigs from American and Chinese chestnut respectively, from pyrosequencing alone, and 34,800 and 48,335 contigs, with the addition of the Sanger sequences for American chestnut and Chinese chestnut, respectively (Table 2). GO annotation using the *Arabidopsis thaliana *proteome as reference showed that the transcriptome of these species covers a wide range of biological processes (Figure 2) suggesting that the cDNA libraries were unbiased and well-suited for studies of development and physiology. The distribution of biological processes of the identified contigs from American and Chinese chestnut (Figure 2) did not show any statistically significant differences (*p*-value > 0.05) [17]. BLASTX alignments to model system proteomes showed that ~ 60% of the transcript contig sequences from the chestnut species studied have strong similarity to predicted proteins in *Arabidopsis thaliana *or *Populus trichocarpa*. Of the contig sequences that did not have any significant matches to *Arabidopsis thaliana *genes, 5 to 6% had a match to *Populus trichocarpa *genes. The remaining contigs (~30%) did not match any sequence in either the *Arabidopsis thaliana *or *Populus trichocarpa *proteomes. We observed a bias toward longer sequences in the contigs with BLASTX alignments to the model proteomes. The distribution of contig length showed that ~85% of sequences without BLASTX alignments to the proteomes of the two model species were short (< 250 nt). In contrast, only about 50% of contigs with good BLASTX hits on the model species proteomes were shorter than 250 nt.

**Figure 3:**
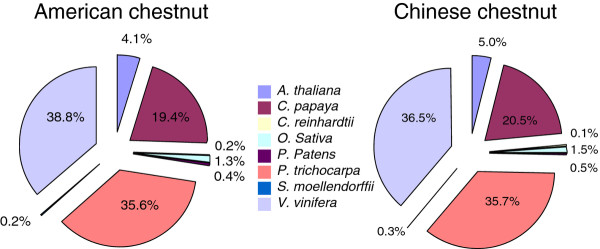
**Comparison of the percentage of American and Chinese chestnut contigs that have best hits on the proteome of each of the model plants**.

BLASTX searches were then conducted against the proteomes of all of the plant species for which the whole genome sequence were available at the time of this study, including *Vitis vinifera*, *Carica papaya*, *Medicago truncatula*, *Oryza sativa*, *Populus trichocarpa*, *Physcomitrella patens*, and *Selaginella moellendorffii*. A large fraction of chestnut contigs had better BLAST alignment scores to woody species than to the herbaceous species (Figure 3). For instance, over 35% of contigs from American and Chinese chestnut had best alignments to *Vitis vinifera *and *Populus trichocarpa*. Only ~5% of the contigs had best alignments to *Arabidopsis thaliana*. This bias cannot be attributed to a GC content difference between contigs from woody versus herbaceous species as their GC content have similar distributions (data not shown).

### Coverage of the transcriptomes

**Figure 4:**
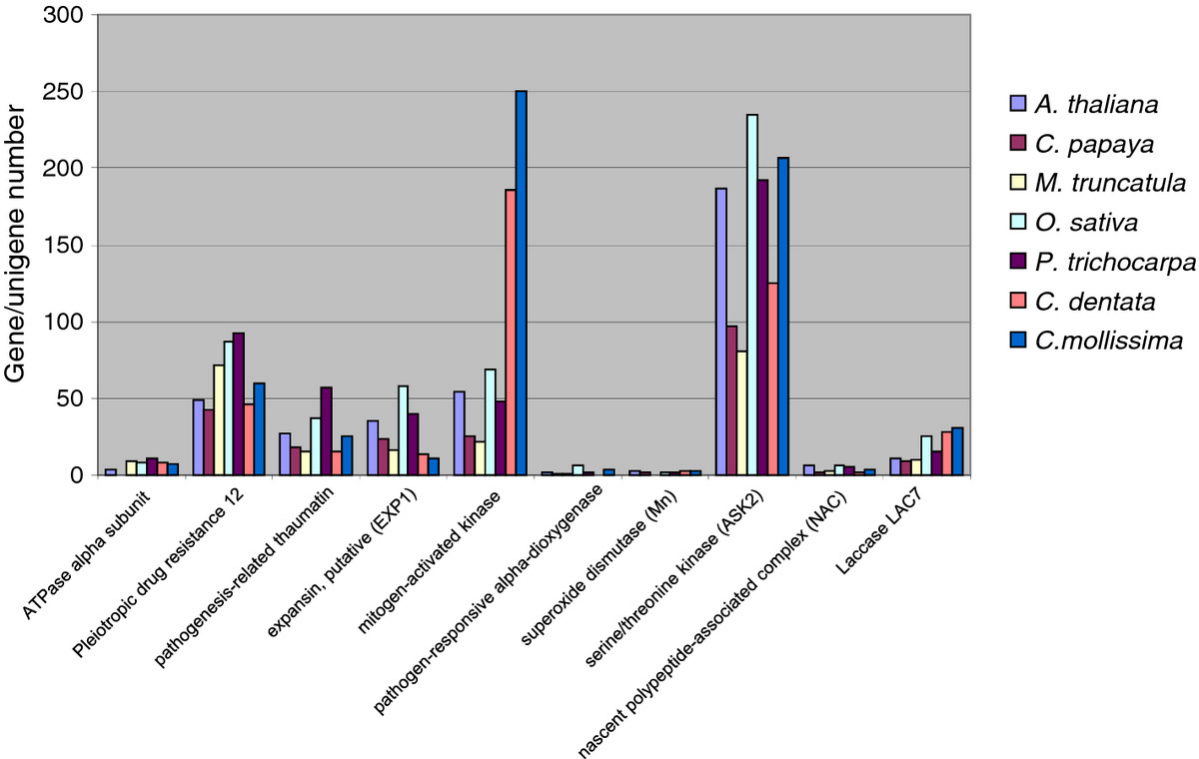
**Size of some defense related gene families in model species**: *Arabidopsis thaliana*, *Populus trichocarpa*, *Oryza sativa*, *Carica papaya*, *Medicago truncatula*, *Castanea molissima*, and *Castanea dentate*.

From the 34,800 and 48,335 contigs, a total of 11,431 and 10,016 large transcript contigs (more than 800 nucleotides) were identified from American chestnut and Chinese chestnut, respectively. The size of the transcript contigs assembled including large ones ranged from 258 bp to 1038 bp with approx. 4-12% of the American and Chinese chestnut contigs covering at least 70% of the length of the coding sequences relative to the respective genes in *Arabidopsis thaliana*. Analyses of the length of contigs showed that 344 (6.0%) and 874 (6.7%) contigs were full length in American chestnut and Chinese chestnut, respectively. Analysis of gene family size, both in American and Chinese chestnut as well as in *Populus trichocarpa*, *Arabidopsis thaliana*, and *Oryza sativa*, showed that the numbers of genes per family identified in chestnuts is similar to their counterpart in the model plant species suggesting a good coverage of the transcriptome in these two Fagaceae species (Figure 4). For instance, the number of members per gene family correlates well between American chestnut, Chinese chestnut, and the other model plant species (Additional file 1: Figure S1), with correlations coefficients of R = 0.8 for Chinese chestnut versus *Arabidopsis *and R = 0.74 for Chinese chestnut versus *Populus*.

### Defense related genes in American and Chinese chestnut

**Figure 5:**
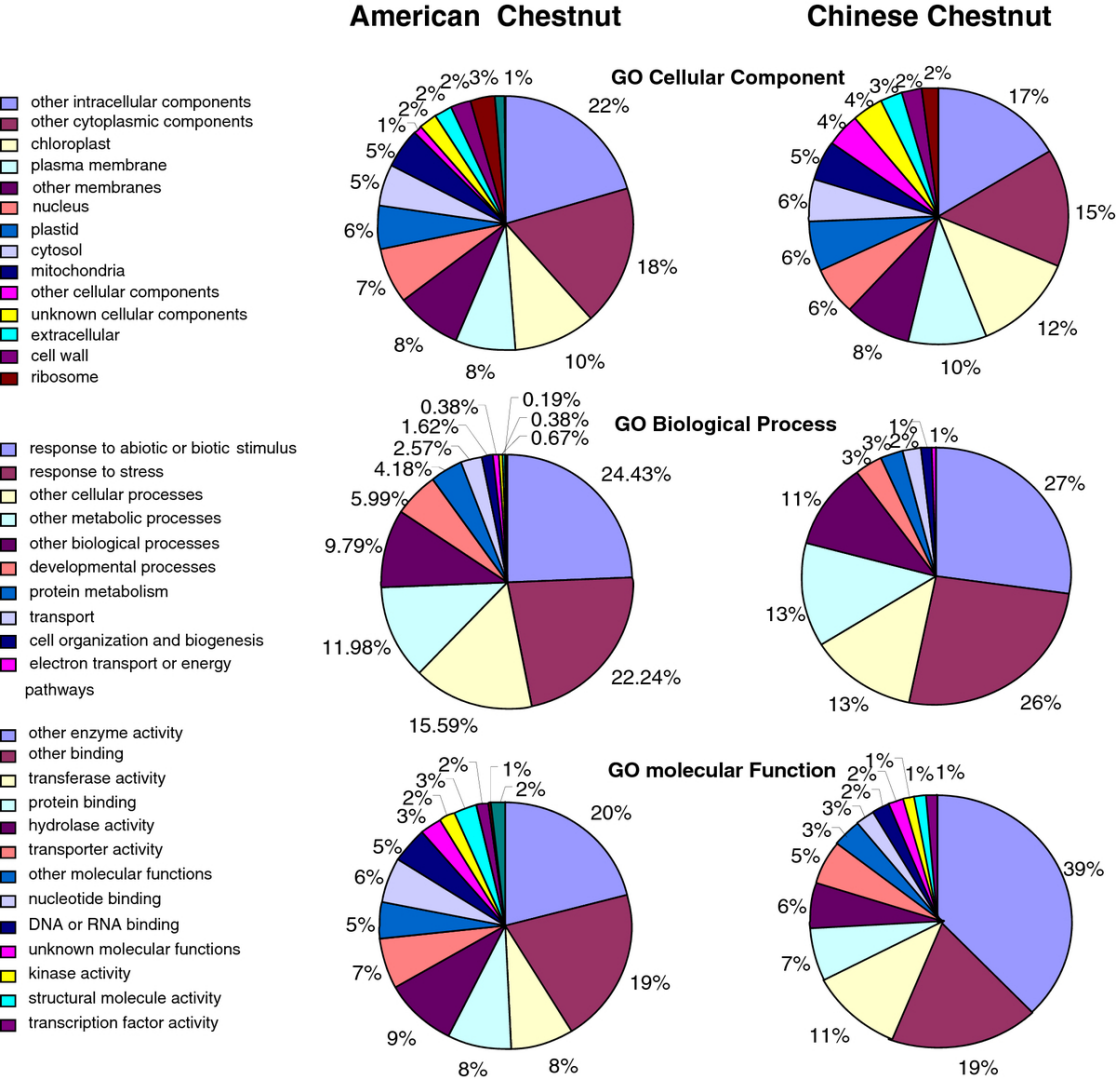
**Pie chart representation of Gene Ontology classification of putative molecular functions of American and Chinese chestnut predicted genes involved in response to biotic stimuli as well as biological processes in which they are involved**.

*In silico *analysis of transcript abundance using the DEGseq approach [16] identified 1715 GDTA in canker tissues versus healthy stems in American chestnut and 720 G DTA in Chinese chestnut (Figure 5, Additional file 2: Table S1, and Additional file 3: Table S2). The number of reads per transcript contig ranged between 7 and 6388 in canker and between 0 and 756 in healthy stem tissues. GO annotation distribution (Figure 5) showed that 177 and 86 of the identified genes from American and Chinese chestnut, respectively, were involved in response to abiotic or biotic stimuli. Twenty two percent and twenty three percent of American chestnut and Chinese chestnut genes from this functional category were involved in defense against biotic stresses. Most of the gene transcripts were highly abundant in canker tissues of both species (Additional file 4: Table S3) and thus represent good candidates for defense against the CBD fungus. GO annotation distribution (Figure 5) showed that the most frequent molecular functions of the identified defense-related genes were hydrolases, protein binding, transferases, and transporters. Several annotation categories including "secondary metabolic process", "oxidoreductase", "cellulose and pectin-containing cell wall", "hydrolases", and "lyases activity" were significantly over-represented in Chinese than American chestnut. A statistical analysis using the GOstat program [18] confirmed the enrichment of Chinese chestnut transcritpome in these functional categories (*p*-value < 0.01). On the opposite, several functional categories including mainly house-keeping genes such "structural constituent of the ribosome", "translation", "ribosome biogenesis and assembly", and "protein metabolic process" were over-represented in American chestnut than Chinese chestnut (*p*-value < 0.01). The over-representation of house-keeping GDTAs in American chestnut could be associated with the increase in protein synthesis at the infection site for defense against the pathogen.

**Figure 6:**
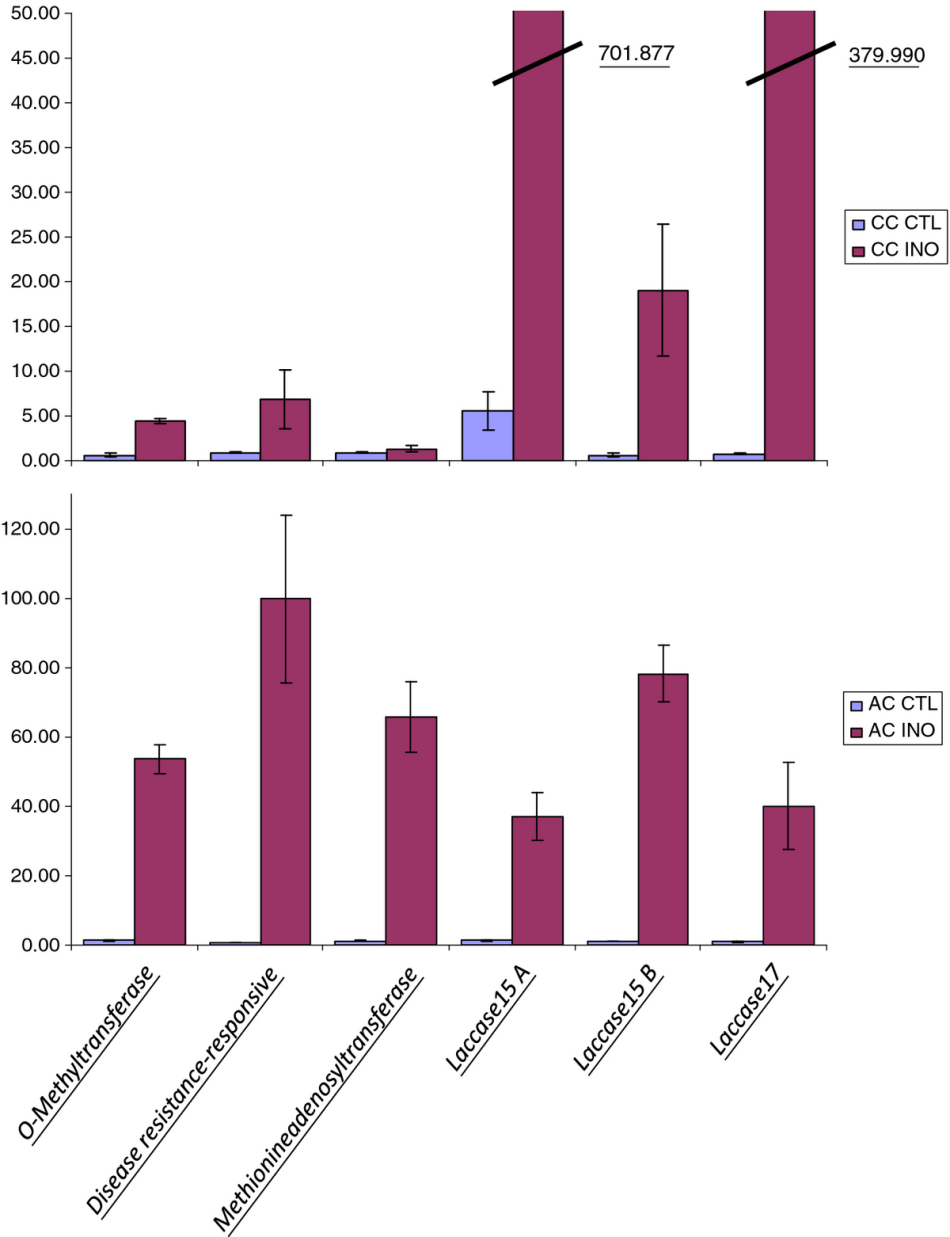
**Expression profiles of defense related genes in *Cryphonectria parasitica *inoculated and control plants in American and Chinese chestnut**. The names of genes studied are indicated on the bottom of diagrams. The tissues analyzed are indicated on the right of the figure. CC CTL, Chinese chestnut control; CC INO, Chinese chestnut inoculated plants; AC CTL, American chestnut control; AC INO, American chestnut inoculated.

The list of the identified defense-related genes showing DTA involves several related pathways (Tables 3 and 4, Additional file 2: Table S1, Additional file 3: Table S2, and Additional file 4: Table S3). The first category includes genes involved in the biosynthesis of lignin and other cell wall components such as *4-coumarate:CoA ligase *(4CL), *Cinnamyl-Alcohol Dehydrogenase *(CAD), *cinnamoyl CoA reductase *(CCR), *peroxidase*, *Myb transcription factor*, and *UDP-glucose:thiohydroximate S-glucosyltransferase*. Genes involved in programmed cell death and hypersensitivity such as *Myo-inositol-1-phosphate*, *ATPase transporter*, *voltage-dependent anion channel*, *2-deoxy-D-arabino-heptulosonate 7-phosphate*, and *cysteine proteinase precursor-like protein *were also identified in canker tissues. However, one of the highly represented categories was phytohormone signaling including ethylene, jasmonic acid (JA), salicyc acid (SA), and abscisic acid (ABA) (Tables 3 and 4, Additional file 1: Figure S1, Additional file 2: Table S1). For example, transcripts of 12 genes involved in JA response were differentially abundant in Chinese chestnut. These include *allene oxide cyclase*, *JAZ1, lipoxygenase*, *12-oxophytodienoate reductase*, *3-ketoacyl-CoA thiolase*, *chitinase*, *plastidic fatty acid desaturase*, and others. Lipooxygenase, chitinase, and ACC oxidase are among genes with the most DTA in canker versus healthy stem (Additional file 3: Table S3). Genes involved in the response to SA include *alpha-dioxygenase*, *mitochondrial chaperonin HSP*, *senescence-associated gene*, and others. Genes related to the ABA response include ABA *8'-hydroxylase*, *26S proteasome regulatory subunit*, *protein phosphatase 2 C*, *hydroxy-2-methyl-2-(E)-butenyl 4-diphosphate (HMBPP) synthase *and others. Several other genes involved were identified such as *MYB transcription factors, proteases*, and *kinases*. Comparison of GDTAs from American and Chinese chestnut showed that similar set of genes were induced in American and Chinese chestnut (Additional file 4: Table S3). Few genes were induced in American versus Chinese and vice versa (Additional file 4: Table S3). Analyses of a small set of these candidate genes using quantitative RT-PCR in healthy stem tissue versus *Cryphonectria parasitica *inoculated stem tissues from American and Chinese chestnut confirmed the differential expression of several of these candidates (Figure 6).

**Table 3:** List of some defense related GDTA in canker versus healthy stem in American chestnut.

Contig name	Ath. BH^1^	# ACC Reads^2^	# ACHS Reads^3^	log2norm	Function
AC454_contig77_v3	AT1G15950.1	58	34	3.19	cinnamoyl CoA reductase.

AC454_contig559_v3	AT1G65060.1	17	0	7.50	isoform of 4-coumarate:CoA ligase (4 cl)

AC454_contig968_v3	AT2G30490.1	11	5	3.55	cinnamate-4-hydroxylase.

AC454_contig15349_v3	AT3G12500.1	49	8	5.03	basic chitinase involved

AC454_contig1339_v3	AT1G74100.1	6	1	5.00	desulfoglucosinolate sulfotransferase

AC454_contig17741_v3	AT3G10920.1	15	25	1.68	manganese superoxide dismutase (MSD1)

AC454_contig14459_v3	AT2G35690.1	59	72	2.13	acyl-CoA oxidase. Involved in jasmonate biosynthesis.

AC454_contig2314_v3	AT1G15520.1	10	1	5.74	abc transporter family

AC454_contig15056_v3	AT2G41430.1	25	69	0.95	hydrophilic protein lacking Cys residues

AC454_contig8521_v3	AT3G60160.1	6	3	3.42	member of MRP subfamily

AC454_contig38515_v3	AT1G80600.1	6	0	6.00	HopW1-1-Interacting protein 1 (WIN1).

AC454_contig1398_v3	AT2G23620.1	9	3	4.00	carboxylesterase/methyl jasmonate esterase

AC454_contig27747_v3	AT5G60600.1	30	65	1.30	hydroxy-2-methyl-2-(E)- butenyl 4-diphosphate synthase

AC454_contig4243_v3	AT4G11260.1	43	142	0.69	SCF(TIR1) mediated degradation of Aux/IAA proteins

AC454_contig117_v3	AT4G37980.1	63	0	9.39	oxidoreductase activity

AC454_contig12110_v3	AT3G60190.1	8	5	3.09	Arabidopsis dynamin-related protein 1E, DRP1E

AC454_contig1301_v3	AT2G26560.1	6	6	2.42	lipid acyl hydrolase with wide substrate specificity

AC454_contig2362_v3	AT3G04720.1	6	0	6.00	similar to the antifungal chitin-binding protein hevein

AC454_contig575_v3	AT1G73260.1	6	0	6.00	trypsin inhibitor

AC454_contig33210_v3	AT3G20600.1	6	4	3.00	non-race specific resistance to bacterial and fungal pathogens

AC454_contig22127_v3	AT4G37870.1	39	101	1.04	putative phosphoenolpyruvate carboxykinase

AC454_contig9688_v3	AT4G23130.1	22	25	2.23	receptor-like protein kinase.

AC454_contig18720_v3	AT4G09320.1	39	32	2.70	nucleoside diphosphate kinase type 1 (NDPK1) gene

AC454_contig7385_v3	AT4G29040.1	24	28	2.19	26S proteasome AAA- ATPase subunit RPT2a (RPT2a)

AC454_contig5037_v3	AT2G18960.1	8	5	3.09	plasma membrane proton ATPase

AC454_contig38744_v3	ATCG00120.1	5	2	3.74	ATPase alpha subunit

AC454_contig16366_v3	AT1G33970.2	6	4	3.00	avirulence-responsive protein

AC454_contig14337_v3	AT1G35720.1	31	78	1.08	a member of the annexin gene family

AC454_contig28989_v3	AT4G11600.1	43	23	3.32	glutathione peroxidase.

AC454_contig1677_v3	AT1G45249.1	5	1	4.74	Leucine zipper transcription factor

AC454_contig1141_v3	AT3G11410.1	4	1	4.42	protein phosphatase 2 C

AC454_contig6419_v3	AT1G69530.1	56	35	3.09	Member of Alpha-expansin Gene Family

AC454_contig1070_v3	AT4G37990.1	5	1	4.74	alcohol:NADP + oxidoreduct ase

AC454_contig12900_v3	AT1G04410.1	48	59	2.12	malate dehydrogenase, cytosolic, putative.

AC454_contig2256_v3	AT4G16260.1	6	4	3.00	catalytic/cation binding /hydrolase

AC454_contig1025_v3	AT3G57240.1	24	0	8.00	glycosyl hydrolase family 17

AC454_contig4431_v3	AT3G25070.1	4	2	3.42	R protein complex

AC454_contig2340_v3	AT5G48485.1	20	31	1.78	putative apoplastic lipid transfer protein

AC454_contig6410_v3	AT1G20030.1	27	10	3.85	pathogenesis-related thaumatin family protein

AC454_contig18835_v3	AT4G38660.2	5	3	3.15	thaumatin, putative

AC454_contig3796_v3	AT5G47390.1	19	48	1.08	myb family transcription factor

AC454_contig23403_v3	AT5G35620.1	14	15	2.32	Cap-binding protein

^1^, Ath. BH: *Arabidopsis *best hit; ^2^, # ACC Reads: number of reads in American chestnut canker libraries; ^3^, # ACHS Reads: number of reads in American chestnut healthy stem libraries

**Table 4:** List of some defense related GDTA in canker versus healthy stem tissues in Chinese chestnut.

Contig Name	Ath. BH^1^	# CCC Reads^2^	# CCHS Reads^3^	log2 norm^4^	Function
CCall_contig18_v2	AT1G02500	68	31	2.33	S-adenosylmethionine synthetase

CCall_contig24687_v2	AT1G02800	29	1	6.05	endo-1,4-beta glucanase (CEL2)

CCall_contig45501_v2	AT1G04410	111	155	0.71	malate dehydrogenase

CCall_contig26701_v2	AT1G05010	260	12	5.63	1-aminocyclopropane-1- carboxylate oxidase

CCall_contig35157_v2	AT1G06180	8	0	5.20	MYB3R- and R2R3- type MYB- encoding genes

CCall_contig28632_v2	AT1G13280	33	9	3.07	allene oxide cyclase

CCall_contig39585_v2	AT1G13440	294	211	1.67	Glyceraldehyde-3-phosphate dehydrogenase C2

CCall_contig27982_v2	AT1G15520	26	8	2.90	ABC transporter family

CCall_contig42826_v2	AT1G15950	66	56	1.43	cinnamoyl CoA reductase

CCall_contig13029_v2	AT1G19180	45	30	1.78	JAZ1 is a nuclear-localized protein

CCall_contig9319_v2	AT1G22450	35	37	1.12	subunit 6b of cytochrome c oxidase

CCall_contig48116_v2	AT1G24020	9	0	5.37	MLP-LIKE PROTEIN 423 (MLP423)

CCall_contig22194_v2	AT1G47128	94	87	1.31	cysteine proteinase precursor- like protein

CCall_contig8876_v2	AT1G51470	8	0	5.20	myrosinase.

CCall_contig35286_v2	AT1G52340	23	7	2.91	cytosolic short-chain dehydrogenase/reductase

CCall_contig38717_v2	AT1G55020	19	0	6.44	lipoxygenase

CCall_contig12135_v2	AT1G58440	15	5	2.78	squalene monooxygenase activity.

CCall_contig30860_v2	AT1G59960	133	155	0.97	aldo/keto reductase

CCall_contig42753_v2	AT1G64520	26	20	1.57	Regulatory Particle non-ATPase 12a (RPN12a)

CCall_contig1090_v2	AT1G65930	80	109	0.75	NADP +- isocitrate dehydrogenase

CCall_contig24182_v2	AT2G06050	25	16	1.84	12-oxophytodienoate reductase

CCall_contig41807_v2	AT2G16500	84	50	1.94	arginine decarboxylase (ADC)

CCall_contig12372_v2	AT2G18950	19	13	1.74	homogentisate phytyltransferase

CCall_contig11468_v2	AT2G22240	171	74	2.40	Myo-inositol-1-phosphate synthase isoform 2

CCall_contig19221_v2	AT2G26710	8	0	5.20	cytochrome p450 family

CCall_contig3763_v2	AT2G30080	10	3	2.93	member of Fe(II) transporter isolog family

CCall_contig43865_v2	AT2G30490	28	16	2.00	cinnamate-4-hydroxylase

CCall_contig3045_v2	AT2G33150	56	71	0.85	organellar 3-ketoacyl-CoA thiolase

CCall_contig28249_v2	AT2G37170	140	194	0.73	plasma membrane intrinsic protein (PIP2)

CCall_contig45019_v2	AT2G38710	14	8	2.00	AMMECR1 family

CCall_contig22956_v2	AT2G38870	39	0	7.48	PR (pathogenesis-related) peptide

CCall_contig28937_v2	AT2G47510	10	2	3.52	FUM1: fumarase

CCall_contig3196_v2	AT3G01280	47	56	0.94	voltage-dependent anion channel

CCall_contig46205_v2	AT3G01420	7	0	5.00	alpha-dioxygenase

CCall_contig28792_v2	AT3G04120	12	1	4.78	cytosolic GADPH (C subunit)

CCall_contig8907_v2	AT3G04720	17	4	3.28	similar to the antifungal chitin- binding

CCall_contig41513_v2	AT3G10920	42	26	1.89	manganese superoxide dismutase (MSD1)

CCall_contig3523_v2	AT3G10985	36	34	1.28	A senescence-associated gene

CCall_contig27333_v2	AT3G12360	39	34	1.39	protein with an ankyrin motif

CCall_contig39986_v2	AT3G12490	39	33	1.44	cysteine proteinase inhibitor activity

CCall_contig42078_v2	AT3G12500	73	13	3.69	basic chitinase

CCall_contig46415_v2	AT3G23600	72	6	4.78	dienelactone hydrolase family protein

CCall_contig11692_v2	AT3G45140	103	63	1.90	Chloroplast lipoxygenase

CCall_contig8863_v2	AT4G01070	8	1	4.20	glycosyltransferase (UGT72B1)

CCall_contig11269_v2	AT4G15480	217	99	2.33	sinapic acid:UDP-glucose glucosyltransferase

CCall_contig38534_v2	AT4G34135	11	2	3.66	flavonol 7-O-glucosyltransferase (EC 2.4.1.237)

CCall_contig18339_v2	AT4G37530	7	1	4.00	peroxidase, putative

CCall_contig4088_v2	AT4G37980	150	15	4.52	ELICITOR-ACTIVATED GENE 3-1 (ELI3-1)

CCall_contig24556_v2	AT4G37990	29	0	7.05	aromatic alcohol:NADP + oxidoreductase

CCall_contig16847_v2	AT4G39980	8	0	5.20	2-deoxy-D-arabino-heptulosonate 7-phosphate synthase

CCall_contig14020_v2	AT5G06950	17	11	1.82	Transcription factor of the B-ZIP family

CCall_contig45136_v2	AT5G14740	147	19	4.15	beta carbonic anhydrase

CCall_contig41707_v2	AT5G42650	184	0	9.72	cytochrome p450 CYP74 gene family

CCall_contig46027_v2	AT5G45340	16	0	6.20	protein with ABA 8'-hydroxylase activity

CCall_contig9507_v2	AT5G52640	22	15	1.75	cytosolic heat shock protein AtHSP90.1

CCall_contig42347_v2	AT5G64250	13	4	2.90	2-nitropropane dioxygenase family /NPD family

^1^, Ath. BH: *Arabidopsis *best hit; ^2^, # CCC Reads: number of reads in Chinese chestnut canker libraries; ^3^, # CCHS Reads: number of reads in Chinese chestnut healthy stem libraries; ^4^, log2 norm, Expression ratio normalized

## Discussion

In this study, over 1.5 million sequencing reads from various tissues were generated and assembled into 34,800 and 48,501 contigs, for American and Chinese chestnut respectively. The low level of contamination with organelle DNA, the fraction of complete or nearly complete full length cDNA sequences, and the depth coverage of genes involved in various biological processes indicate that pyrosequencing is an excellent tool for gene discovery, EST sequencing, and transcriptome analysis in non-model tree species. Combining 454 and Sanger sequences can improve contig construction as the assembly using both data sets resulted in more reads being integrated into contigs than by assembly of pyrosequences alone, resulting in slightly fewer but longer contig sequences. Using a tool developed recently [19], that estimates the amount of sequencing needed to cover the transcriptome of a given species, that takes into consideration the sequencing platform and the number of contigs generated from each species, we determined that as few as two or three additional plates of 454 sequence from different developmental stages and physiological conditions should allow for 100% coverage of the transcriptomes of Chinese and American chestnut.

The sequencing effort of the GTDF increased the number of Fagaceae cDNAs available in GenBank from a few sequences to hundreds of thousands of sequences for each of the species studied. The cDNA sequences and contig sequences, which are publicly accessible at the website [14] hosted at Clemson University, provides tools for Fagaceae species-specific BLAST searches. The cDNA sequences have been used to select molecular markers such as Simple Sequence Repeats (SSRs), and Single Nucleotide Polymorphisms (SNPs) for genetic mapping in Chinese and American chestnut (Kubisiak et al., in preparation), for ordering the physical map of Chinese chestnut (Fang et al., in preparation), and for characterizing microRNAs (Barakat et al., unpublished data). The sequences were also used to identify candidate genes involved in resistance to CBD [15], some of which are being functionally characterized (Powell et al., unpublished data). The genes with DTA were also being examined for colocalization with QTLs for resistance to CBD.

BLASTX alignments to the proteomes of two model systems (*Arabidopsis *or *Populus*) showed that ~ 60% of the transcript contig sequences from the Fagaceae species studied have strong similarity to predicted proteins. The remaining contigs did not match any sequence in the *Arabidopsis *or *Populus *proteomes. A large fraction of these are short sequences that may originate from 3' or 5' untranslated regions, which tend to be highly divergent between species (Additional file 1: Figure S1). Part of these sequences may also correspond to non-coding RNAs, or potential chestnut-specific genes. BLAST searches against the proteomes of eight plant species with complete genome sequences (*Vitis vinifera*, *Carica papaya*, *Medicago truncatula*, *Oryza sativa*, *Populus trichocarpa*, *Selaginella moellendorffii*, *Physcomitrella patens*, and *Chlamydomonas reinhardtii*) showed that a large fraction of the Fagaceae contigs have better alignments with genes in woody species such as *Vitis vinifera*, *Populus trichocarpa*, *and Carica papaya versus *the non-woody species angiosperm species. Similar results have been reported for other tree species such as *Liriodendron tulipifera *and *Carica papaya *[20,21]. Many long-lived woody plants, including Fagaceae species, exhibit extended phases of juvenile development before they reach flowering age, whereas most herbaceous plants reach flowering age in a single growing season. Thus, these observations could be associated with a slower evolutionary rate of genes in woody species versus herbaceous species.

*In silico *analysis of gene expression identified over two fold GDTAs in American than Chinese chestnut. However, most of the difference between the two DGTA sets lies in the number of induced housekeeping genes associated with the increase in resource utilization for plant defense at the infection site. The difference in the number of house-keeping genes induced in American and Chinese is maybe due to the amplitude of response of the two species to *C. parasitica *infection. The number of genes belonging to the category "response to biotic and abiotic stimuli" in American and Chinese chestnut from this is over 14 and 6 times higher than the ones reported previously using a partial dataset [22]. Also, several genes identified previously were not confirmed in this analysis. However, many new genes with DTA were identified in this study. The discrepancy between these results is linked to the larger dataset, which includes both 454 and capillary sequences, the better contig assembly, and the use of a more powerful tool for DTA analysis.

Genes with DTA identified in this study belong to well known plant pathways such as phenylpropanoid metabolism, phytohormone (JA, ABA, ethylene and SA) signaling, cell wall biosynthesis, proteolysis, and others. These genes and pathways function at different times in the plant response to pathogens. The category of genes involved in phenylpropanoid metabolism act early in plant defense, serving to inhibit or to block the penetration and the progression of the plant pathogen. This category includes genes for biosynthesis of monolignol and other phenolic compounds. Previous studies [23-27] showed that lignin biosynthesis is crucial for cell wall apposition, one of the first lines of plant defense against invading fungi. Besides lignin, the biosynthesis of other polymers such as callose seems to follow infection as suggested by the increased transcript abundance of UDP*-glucose:thiohydroximate S-glucosyltransferase *[28]. Other phenolic products that are involved in plant defense against pest and pathogens seem to be produced as well, as deduced by the presence of transcripts encoding genes such as *flavanone 3-hydroxylase *and *flavonol 7-O-glucosyltransferase *known to regulate flavonoid biosynthesis [29].

The second most important category of genes detected in response to the blight infection includes genes from phytohormone signaling pathways including JA, SA, and ethylene. These hormones trigger the activation of induced systemic resistance and systemic acquired resistance (SAR) to nectrotrophic pathogens [30,31]. The SAR is an effective defense mechanism against a broad range of pathogens and insects. Several genes from the JA response pathway such as *methyl jasmonate esterase (MES1)*, *acyl-CoA oxidase*, *a phyB pathway*, and *ATPase transporter *were identified [32]. Genes involved in SA response such as hydroxy-2-methyl-2-(E)-butenyl 4-diphosphate, HopW1-1-Interacting protein 1 (WIN1) were identified [33]. The SA pathway, which is considered one of the major pathways involved in defense against nectrotrophic pathogens, regulates the expression of defense effector genes and systemic acquired resistance through the repression of the auxin signaling pathway [16,33-36]. Another hormone that seems to play a role in the resistance of chestnut to CBD is abscissic acid (ABA). While ABA was described as a susceptibility factor, other studies [37,38] showed that it activates plant defense by priming for callose deposition or by restricting the progression of the fungus *Cochliobolus miyabeanus *in the mesophyll of rice [38]. Other signaling genes involved in SAR that induce numerous defense genes include apoplastic lipid transfer protein, and basic chitinase, etc [39].

The third category of genes with DTA in canker tissues includes genes involved in early response as part of the HR. Among these are transcripts encoding proteins such as ATPase transporter, kinases, carbonic anhydrase, AMMECR1, MIPS1, voltage-dependent anion channel, 2-deoxy-D-arabino-heptulosonate 7-phosphate (DAHP) synthase, and glutathione peroxidase that were reported previously to be involved in the hypersensitivity resistance (HR) and cell death in plants under pathogenic attack [18,31,40-43]. Reactive oxygen species (ROS) seem to be induced following *C. parasitica *infection as several genes involved in oxidative stress (*alpha-dioxygenase, fumarase*, *cytosolic GADPH (C subunit)*, *cytosolic ascorbate peroxidase APX1*) had more abundant transcripts. Furthermore, several pathogenesis related (PR) genes such as *elicitor-activated gene 3-1 *(*ELI3-1*), *aromatic alcohol:NADP + oxidoreductase*, *thaumatin, pathogenesis-related*, and *antifungal chitin-binding protein *had differentially abundant transcripts in canker versus healthy stem tissues. PR proteins, of which some have antimicrobial functions [44], are mainly induced in localized pathogen attack around HR lesions. It is unknown what roles the HR and cell death play for chestnut defense against a necrotrophic pathogen such as *C. parasitica*. Alternatively, some of the genes involved in the HR may activate a systemic response of the plant or the pathogen may trigger HR to facilitate its colonization of the plant as reported for other pathogens [45]. Several other genes involved in defense such as eIF(iso)4E [46] were also implicated.

The candidate genes identified in this study represent a valuable resource for studying the genetic basis underlying resistance to CBD and the isolation of the fungal pathogen resistance genes. Comparative mapping of the blight resistance quantitative trait loci (QTL) of chestnut with peach disease resistance QTL is revealing that the genes for several of the differentially abundant chestnut transcripts in canker versus healthy stem tissues map to disease resistance QTL regions in both species (Fang and collaborators, in preparation). This suggests that some of the genes identified in this study may play a major role in plant defense against the CBD. Because most of the defense genes and gene networks were induced in both American and Chinese chestnut canker tissues, the question that remains is what then accounts for one of these species being susceptible and the other resistant to CBD. The timing of the response to the pathogen infection, and the amplitude of the response, could result in Chinese chestnut resistance to the blight. More information from transcript profiling during the time course of infection in these two species is required to address this question.

## Conclusions

This project has generated over 83,000 transcript contigs from American and Chinese chestnuts and identified hundreds of genes and regulation pathways which may be involved in chestnut resistance to the pathogen *C. parasitica*. These resources have also been used for SNP and SSR marker development, genetic mapping, physical mapping, and genome organization comparison among chestnut species and between chestnut and other closely related species. Several of the candidate genes identified in this study are in the process of being analyzed for their function using transformation *in planta*. The cDNA database generated in this project is also being used to map expressed genes and to annotate the proteome in the Chinese chestnut genome that is being assembled during submission of this report (John Carlson, personal communication).

## Methods

### Plant materials

Ten cDNA libraries, representing a range of tissues from American and Chinese chestnut were prepared for EST sequencing by 454 technology [47] (Table 1). Tissue samples were submerged in liquid nitrogen immediately upon collection and stored at - 80°C until use. To create cankers, the stems of chestnut trees were inoculated with the hypervirulent *C*. *parasitica *strain EP155 as described by Hebard and collaborators [15,48]. For American chestnut and Chinese chestnut, canker tissue was collected at 5 and 14 days post-inoculation as previously described [15,48]. These times correspond to early and late stages of interaction between the plant and the pathogen [48].

### RNA preparation, cDNA library synthesis, and 454 sequencing

Total RNA was prepared as described previously [49], and assessed with a 2100 Bioanalyzer (Agilent Technologies). cDNA and 454 libraries were constructed as described [15]. The 454 libraries were sequenced using the model GS20, for the American chestnut canker and Chinese chestnut canker libraries, and an FLX model 454 DNA sequencer (Roche Diagnostics), as previously described [15], for the remaining libraries. The sequence data was deposited into the Short Read Archive at the National Center for Biotechnology Information (study accession SRP000395).

### Sanger sequencing

To analyze the transcriptomes of American and Chinese chestnut, this project generated Sanger sequences for about ~9000 cDNA clones from a subtractive library enriched in genes highly expressed in canker tissues in Chinese versus American chestnut using capillary sequencing. A total of 8,101 cDNA sequences were obtained after filtering reads for quality. RNA was prepared using the method described previously [49] and reverse transcribed using the SMART PCR cDNA Synthesis Kit (Clontech, Mountain View, CA). Substrative libraries were constructed using Chinese chestnut as the tester and American chestnut as the driver following the manufacturer recommendation (Clontech). Sequencing of the substractive libraries was conducted at the Clemson University Genomics Institute by an automated Sanger sequencing protocol.

### Transcript assembly and contig annotation

The 454 sequence reads were assembled into contigs using 454 Newbler (Roche Diagnostics) or SeqMan™ NGen™ v1.2 software (DNAStar, Inc), optimized for 454 next generation data. The new assemblies are available on the Fagaceae website [14]. cDNA libraries were constructed using random priming which results in low poly A/T tail contamination and therefore no filtering was performed. Also, SeqMan removes low quality ends including homopolymer runs of poly(A/T) that have lower qualities in 454 sequencing. Contamination with mitochondrial and chloroplast genes was assessed by running a BLASTX search against *Arabidopsis *mitochondrion and chloroplast proteomes. An assembly using 454 and 9000 Sanger sequences was performed and compared the one that used 454 sequences only. Full-length contigs were identified by running a BLASTX search against the *Arabidopsis thaliana *proteome and comparing the lengths of the aligned portion of each contig and the putative proteins [50]. The annotation of contigs was performed by BLASTX [51] against the *Arabidopsis thaliana *proteome (e-value = e^-5^) and the Gene Ontology (GO) [52] system [15]. Comparison of GO annotation distribution between species was conducted using the GOstat program [17] set to the following parameters: GO-DB: tair; Min Sub-GO length: 3; *P*-Value Cutoff: 0.01; GO-Cluster Cutoff: -1; with no correction for multiple testing because the high dependence between GO terms will cause the test to be overly conservative. To determine which model species with most best hits to Fagaceae transcript contigs, BLAST alignments were conducted by querying the Fagaceae contigs against the proteomes of algal, moss and higher plant species with fully sequenced genomes (*Chlamydomonas reinhardtii*, *Physcomitrella patens*, *Selaginella moellendorffii*, *Oryza sativa*, *Vitis vinifera*, *Populus trichocarpa*, *Carica papaya*, and *Arabidopsis thaliana*) and the e-values of the best hits from each species were compared.

### Identification of DTA in canker tissues

DEGseq [16] was used to identify gene specific differences in transcript abundance. The DEGseq package was chosen because it integrates several statistical methods, can estimate a theoretical replicate when an experimental one is not provided, and has been used routinely to identify DTA [16,53,54]. The number of 454 reads per contig for each gene was compared between canker and healthy stem tissues in American and Chinese chestnut separately. Similar analyses were performed for gene orthologs from both species. Orthologs were identified using a reciprocal best hit approach. DEGseq employs a random sampling model based on the read count in canker and healthy stem tissue libraries and performs a hypothesis test based on that model. Two theoretical four-fold local standard deviation lines can be drawn on the expression MA-plot to estimate the noise level of genes with different intensities and identify gene expression differences in different libraries. Genes passing the threshold are identified as exhibiting DTA. GO enrichment analyses were performed using Blast2Go software [55].

### Validation tests of GDTA by real-time quantitative RT-PCR

Real-time quantitative RT-PCR tests were conducted to determine the extent to which the number of EST reads per gene obtained by shotgun sequencing accurately reflected transcript levels in the source tissues. RT-PCR estimates of transcript abundance were conducted on RNA from healthy and canker stem tissues from American chestnut and Chinese chestnut. RT-PCRs were performed as described previously [56,57]. Quantitative real time PCRs (qRT-PCRs) were prepared using the SYBR Green Master Mix kit (Applied Biosystems) and run in an Applied Biosystems 7500 Fast Real-Time PCR system with default parameters. Primers were designed using Primer Express^® ^software (Applied Biosystems). A gene encoding 18S rRNA was used as an endogenous standard to normalize template quantity. We used only one standard because we did not observe any tissue specific differences in expression of *18S rRNA *gene in our study. In addition, RT-PCR analyses were performed to confirm the expression of GDTA already identified using *in silico *expression analysis. For each gene, three biological replicates (three different trees) and three technical replicates were performed. Statistical analyses used Statistica 6.0 software (StatSoft Poland Inc., Tulsa, OH, USA), to estimate the significance of the differences.

## Abbreviations

CBD: Chestnut blight disease; cDNA: Complementary DNA; EST: Expressed sequence tag; DTA: Differential transcript abundance; GTDF: Genomic tool development for the Fagaceae; Mya: Million years ago; PCD: Programmed cell death; QTL: Quantitative trait locus; SNP: Single nucleotide polymorphism; SSR: Simple sequence repeat; JA: Jasmonic acid; SA: Salicylic acid; ABA: Abscisic acid.

## Competing interests

The authors declare that they have no competing interests

## Authors' contributions

AB conceived and performed the transcriptome study, analyzed the DEG results, performed the RT-PCR experiments, wrote the manuscript, and supervised the work of NBMY, JP, and CY. NBMY, JP and CY contributed to the RT-PCR analyses and helped prepare the figures and Tables. MS, SF, and CC assembled the 454 cDNA reads and run the DEG analysis. FH performed the chestnut inoculation experiments and provided canker and healthy stem tissues. BP and KB constructed the cDNA subtractive libraries. SS conducted 454 library sequencing. NW, BP, JEC, AA, and RS designed the 454 library construction, provided advice and oversight on sequencing, and edited the manuscript. All authors read and approved the final manuscript.

## Supplementary Material

Additional file 1 **Correlation between the number of genes per gene family in American chestnut, Chinese chestnut, *Arabidopsis*, and *Populus***. Click here for file

Additional file 2 **List of genes with differential accumulated transcripts in canker tissue versus healthy stem in American chestnut**. Click here for file

Additional file 3 **List of genes with differential accumulated transcripts in canker versus healthy stem tissues in Chinese chestnut**. Click here for file

Additional file 4 **List of genes with differential accumulated transcripts in infected tissues of Chinese chestnut (CC) and American chestnut (AC), or in one species but not in the other**. Click here for file

## References

[B1] Flora of North America http://www.efloras.org

[B2] Oh S Manos PS Molecular phylogenetics and cupule evolution in Fagaceae as inferred from nuclear CRABS CLAW sequences Taxon 2008 57 434 451

[B3] Anagnostakis S The effect of multiple importations of pests and pathogens on a native tree Biological Invasions 2001 3 245 254 10.1023/A:1015205005751

[B4] Hill J Wildlife value of Castanea dentata past and present, the historical decline of the chestnut and its future use in restoration of natural areas Proceedings of the International Chestnut Conference: 1994; Morgantown, West. Virginia 1994 West Virginia University Press 186 193

[B5] Herendeen PS Magallon-Puebla S Lupia R Crane PR Kobylinska J A preliminary conspectus of the Allon Flora from the Late Cretaceous (Late Santonian) of central Georgia, U.S.A Ann Missouri Bot Garden 1999 86 407 471 10.2307/2666182

[B6] Wang H Moore MJ Soltis PS Bell CD Brockington SF Alexandre R Davis CC Latvis M Manchester SR Soltis DE Rosid radiation and the rapid rise of angiosperm-dominated forests Proc Natl Acad Sci USA 2009 106 10 3853 3858 10.1073/pnas.0813376106 19223592 PMC2644257

[B7] Sederoff R Myburg A Kirst M Genomics, domestication, and evolution of forest trees Cold Spring Harb Symp Quant Biol 2009 74 303 317 10.1101/sqb.2009.74.040 20375318

[B8] Griffin GJ Blight Control and Resoration of the American Chestnut J Forestry 2000 99 22 27

[B9] Andrade GM Merkle SA Enhancement of American chestnut somatic seedling production Plant Cell Rep 2005 24 6 326 334 10.1007/s00299-005-0941-0 15789206

[B10] The American Chestnut Foundation http://www.acf.org

[B11] Hebard FV The backcross breeding program of the American chestnut foundation Restoration of American chestnut to forest lands--Proc of a conference and workshop: 2006; The North Carolina Arboretum 2006 National Park Service Natural Res Rep NPS/NCR/CUE/NRR-2006/001

[B12] Milgroom MG Cortesi P Biological control of chestnut blight with hypovirulence: a critical analysis Annu Rev Phytopathol 2004 42 311 338 10.1146/annurev.phyto.42.040803.140325 15283669

[B13] Wheeler N Sederoff R Role of genomics in the potential restoration of the American chestnut Tree Genetics Genomes 2008 5 181 187

[B14] The Fagaceae Genomic Tool Project http://www.fagaceae.org

[B15] Barakat A DiLoreto D Zhang Y Smith C Baier K Powell W Wheeler N Sederoff R Carlson J Comparison of the transcriptomes of American chestnut (Castanea dentata) and Chinese chestnut (Castanea mollissima) in response to the chestnut blight infection BMC Plant Biology 2009 9 1 51 10.1186/1471-2229-9-51 19426529 PMC2688492

[B16] Wang L Feng Z Wang X Zhang X DEGseq: an R package for identifying differentially expressed genes from RNA-seq data Bioinformatics 2010 26 1 136 138 10.1093/bioinformatics/btp612 19855105

[B17] Beissbarth T Speed TP GOstat: find statistically overrepresented Gene Ontologies within a group of genes Bioinformatics 2004 20 9 1464 1465 10.1093/bioinformatics/bth088 14962934

[B18] Bovie C Ongena M Thonart P Dommes J Cloning and expression analysis of cDNAs corresponding to genes activated in cucumber showing systemic acquired resistance after BTH treatment BMC Plant Biol 2004 4 15 10.1186/1471-2229-4-15 15331019 PMC516775

[B19] Wall PK Leebens-Mack J Muller KF Field D Altman NS de Pamphilis CW PlantTribes: a gene and gene family resource for comparative genomics in plants Nucleic Acids Res 2008 36 Database D970 D976 18073194 10.1093/nar/gkm972 PMC2238917

[B20] Liang H Ayyampalayam S Wickett N Barakat A Xu Y Landherr L Ralph P Jiao Y Xu T Schlarbaum S Generation of a large-scale genomic resource for functional and comparative genomics in *Liriodendron tulipifera *L Tree Genetics & Genomes 2011 7 5 941 954 10.1007/s11295-011-0386-2

[B21] Ming R Hou S Feng Y Yu Q Dionne-Laporte A Saw JH Senin P Wang W Ly BV Lewis KL The draft genome of the transgenic tropical fruit tree papaya (Carica papaya Linnaeus) Nature 2008 452 7190 991 996 10.1038/nature06856 18432245 PMC2836516

[B22] Barakat A DiLoreto DS Zhang Y Smith C Baier K Powell WA Wheeler N Sederoff R Carlson JE Comparison of the transcriptomes of American chestnut (Castanea dentata) and Chinese chestnut (Castanea mollissima) in response to the chestnut blight infection BMC Plant Biol 2009 9 51 10.1186/1471-2229-9-51 19426529 PMC2688492

[B23] Sibout R Eudes A Mouille G Pollet B Lapierre C Jouanin L Seguin A CINNAMYL ALCOHOL DEHYDROGENASE-C and -D are the primary genes involved in lignin biosynthesis in the floral stem of *Arabidopsis* Plant Cell 2005 17 7 2059 2076 10.1105/tpc.105.030767 15937231 PMC1167552

[B24] Qi X Bakht S Qin B Leggett M Hemmings A Mellon F Eagles J Werck-Reichhart D Schaller H Lesot A A different function for a member of an ancient and highly conserved cytochrome P450 family: from essential sterols to plant defense Proc Natl Acad Sci USA 2006 103 49 18848 18853 10.1073/pnas.0607849103 17124172 PMC1656972

[B25] Kawasaki T Hisako K Nakatsubo T Hasegawa K Wakabayashi K Takahashi H Umemura K Umezawa T Shimamoto K Cinnamoyl-CoA reductase, a key enzyme in lignin biosynthesis, is an effector of small GTPase Rac in defense signaling in rice PNAS 2006 103 230 235 10.1073/pnas.0509875103 16380417 PMC1325009

[B26] Kawalleck P Plesch G Hahlbrock K Somssich IE Induction by fungal elicitor of S-adenosyl-L-methionine synthetase and S-adenosyl-L-homocysteine hydrolase mRNAs in cultured cells and leaves of Petroselinum crispum Proc Natl Acad Sci USA 1992 89 10 4713 4717 10.1073/pnas.89.10.4713 1374911 PMC49153

[B27] Wang G Ding X Yuan M Qiu D Li X Xu C Wang S Dual function of rice OsDR8 gene in disease resistance and thiamine accumulation Plant Mol Biol 2006 60 3 437 449 10.1007/s11103-005-4770-x 16514565

[B28] Clay NK Adio AM Denoux C Jander G Ausubel FM Glucosinolate metabolites required for an *Arabidopsis *innate immune response Science 2009 323 5910 95 101 10.1126/science.1164627 19095898 PMC2630859

[B29] Nicholson RL Hammerschmidt R Phenolic compounds and their role in disease resistance Annu Rev Phytopathol 1992 30 369 389 10.1146/annurev.py.30.090192.002101

[B30] Feys BJ Parker JE Interplay of signaling pathways in plant disease resistance Trends Genet 2000 16 10 449 455 10.1016/S0168-9525(00)02107-7 11050331

[B31] Glazebrook J Contrasting mechanisms of defense against biotrophic and necrotrophic pathogens Annu Rev Phytopathol 2005 43 205 227 10.1146/annurev.phyto.43.040204.135923 16078883

[B32] Vlot AC Liu PP Cameron RK Park SW Yang Y Kumar D Zhou F Padukkavidana T Gustafsson C Pichersky E Identification of likely orthologs of tobacco salicylic acid-binding protein 2 and their role in systemic acquired resistance in *Arabidopsis **thaliana* Plant J 2008 56 3 445 456 10.1111/j.1365-313X.2008.03618.x 18643994

[B33] Lee MW Jelenska J Greenberg JT *Arabidopsis *proteins important for modulating defense responses to *Pseudomonas syringae *that secrete HopW1-1 Plant J 2008 54 3 452 465 10.1111/j.1365-313X.2008.03439.x 18266921

[B34] Rajjou L Belghazi M Huguet R Robin C Moreau A Job C Job D Proteomic investigation of the effect of salicylic acid on *Arabidopsis *seed germination and establishment of early defense mechanisms Plant Physiol 2006 141 3 910 923 10.1104/pp.106.082057 16679420 PMC1489900

[B35] Kiba A Nishihara M Tsukatani N Nakatsuka T Kato Y Yamamura S A peroxiredoxin Q homolog from gentians is involved in both resistance against fungal disease and oxidative stress Plant Cell Physiol 2005 46 6 1007 1015 10.1093/pcp/pci109 15840643

[B36] Gil MJ Coego A Mauch-Mani B Jorda L Vera P The *Arabidopsis *csb3 mutant reveals a regulatory link between salicylic acid-mediated disease resistance and the methyl-erythritol 4-phosphate pathway Plant J 2005 44 1 155 166 10.1111/j.1365-313X.2005.02517.x 16167903

[B37] Wiese J Kranz T Schubert S Induction of pathogen resistance in barley by abiotic stress Plant Biol (Stuttg) 2004 6 5 529 536 10.1055/s-2004-821176 15375723

[B38] De Vleesschauwer D Yang Y Cruz CV Hofte M Abscisic acid-induced resistance against the brown spot pathogen Cochliobolus miyabeanus in rice involves MAP kinase-mediated repression of ethylene signaling Plant Physiol 2010 152 4 2036 2052 10.1104/pp.109.152702 20130100 PMC2850001

[B39] Zander M La Camera S Lamotte O Metraux JP Gatz C *Arabidopsis thaliana *class-II TGA transcription factors are essential activators of jasmonic acid/ethylene-induced defense responses Plant J 2010 61 2 200 210 19832945 10.1111/j.1365-313X.2009.04044.x

[B40] La Camera S Balague C Gobel C Geoffroy P Legrand M Feussner I Roby D Heitz T The *Arabidopsis *patatin-like protein 2 (PLP2) plays an essential role in cell death execution and differentially affects biosynthesis of oxylipins and resistance to pathogens Mol Plant Microbe Interact 2009 22 4 469 481 10.1094/MPMI-22-4-0469 19271961

[B41] Chen K Du L Chen Z Sensitization of defense responses and activation of programmed cell death by a pathogen-induced receptor-like protein kinase in *Arabidopsis* Plant Mol Biol 2003 53 1-2 61 74 14756307 10.1023/B:PLAN.0000009265.72567.58

[B42] Li Y Zou J Li M Bilgin DD Vodkin LO Hartman GL Clough SJ Soybean defense responses to the soybean aphid New Phytol 2008 179 1 185 195 10.1111/j.1469-8137.2008.02443.x 18422900

[B43] Pennell RI Lamb C Programmed Cell Death in Plants Plant Cell 1997 9 7 1157 1168 10.1105/tpc.9.7.1157 12237381 PMC156988

[B44] Sels J Mathys J De Coninck BM Cammue BP De Bolle MF Plant pathogenesis-related (PR) proteins: a focus on PR peptides Plant Physiol Biochem 2008 46 11 941 950 10.1016/j.plaphy.2008.06.011 18674922

[B45] Govrin EM Levine A The hypersensitive response facilitates plant infection by the necrotrophic pathogen Botrytis cinerea Curr Biol 2000 10 13 751 757 10.1016/S0960-9822(00)00560-1 10898976

[B46] Miyoshi H Okade H Muto S Suehiro N Nakashima H Tomoo K Natsuaki T Turnip mosaic virus VPg interacts with *Arabidopsis thaliana *eIF(iso)4E and inhibits in vitro translation Biochimie 2008 90 10 1427 1434 10.1016/j.biochi.2008.03.013 18582528

[B47] Margulies M Egholm M Altman WE Attiya S Bader JS Bemben LA Berka J Braverman MS Chen YJ Chen Z Genome sequencing in microfabricated high-density picolitre reactors Nature 2005 437 7057 376 380 16056220 10.1038/nature03959 PMC1464427

[B48] Hebard F Griffin G Elkins J Developmental histopathology of cankers incited by virulent and hypovirulent *Endothia parasitica *on susceptible and resistant chestnut trees Phytopathology 1984 74 140 149 10.1094/Phyto-74-140

[B49] Chang S Puryear J Cairney J A simple and efficient method for isolating RNA from pine trees Plant Mol Biol Rep 1993 11 113 116 10.1007/BF02670468

[B50] Wall PK Leebens-Mack J Chanderbali AS Barakat A Wolcott E Liang H Landherr L Tomsho LP Hu Y Carlson JE Comparison of next generation sequencing technologies for transcriptome characterization BMC Genomics 2009 10 347 10.1186/1471-2164-10-347 19646272 PMC2907694

[B51] Altschul SF Gish W Miller W Myers EW Lipman DJ Basic local alignment search tool J Mol Biol 1990 215 3 403 410 2231712 10.1016/S0022-2836(05)80360-2

[B52] Consortium GO The Gene Ontology project in 2008 Nucleic Acids Res 2008 36 D440 D444 17984083 10.1093/nar/gkm883 PMC2238979

[B53] Alsford S Turner DJ Obado SO Sanchez-Flores A Glover L Berriman M Hertz-Fowler C Horn D High-throughput phenotyping using parallel sequencing of RNA interference targets in the African trypanosome Genome Res 2011 21 6 915 924 10.1101/gr.115089.110 21363968 PMC3106324

[B54] Chen ZF Matsumura K Wang H Arellano SM Yan X Alam I Archer JA Bajic VB Qian PY Toward an understanding of the molecular mechanisms of barnacle larval settlement: a comparative transcriptomic approach PLoS One 2011 6 7 e22913 10.1371/journal.pone.0022913 21829555 PMC3146488

[B55] Gotz S Garcia-Gomez JM Terol J Williams TD Nagaraj SH Nueda MJ Robles M Talon M Dopazo J Conesa A High-throughput functional annotation and data mining with the Blast2GO suite Nucleic Acids Res 2008 36 10 3420 3435 10.1093/nar/gkn176 18445632 PMC2425479

[B56] Barakat A Bagniewska-Zadworna A Frost CJ Carlson JE Phylogeny and expression profiling of CAD and CAD-like genes in hybrid Populus (P. deltoides × P. nigra): evidence from herbivore damage for subfunctionalization and functional divergence BMC Plant Biol 2010 10 100 10.1186/1471-2229-10-100 20509918 PMC2887455

[B57] Barakat A Bagniewska-Zadworna A Choi A Plakkat U DiLoreto DS Yellanki P Carlson JE The cinnamyl alcohol dehydrogenase gene family in Populus: phylogeny, organization, and expression BMC Plant Biol 2009 9 26 10.1186/1471-2229-9-26 19267902 PMC2662859

