# Supplementary figures and images for: Chestnut resistance to the blight disease: insights from transcriptome analysis
Source: BMC Plant Biol. 2012 Mar 19;12:38. doi: 10.1186/1471-2229-12-38 (PMC3376029; doi:10.1186/1471-2229-12-38)

Additional figure 1

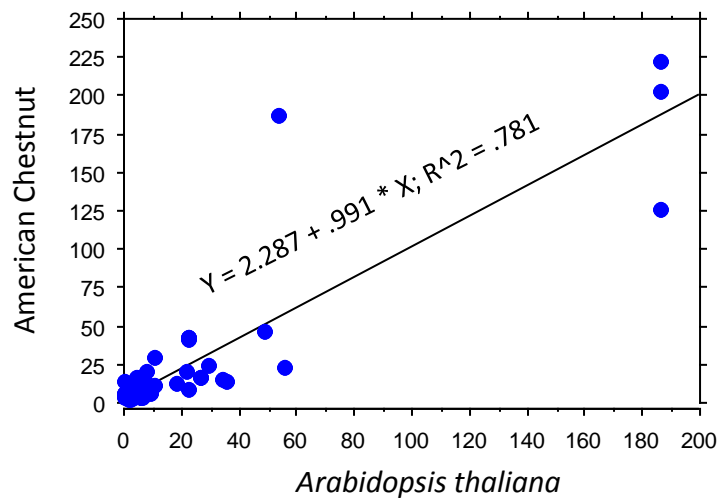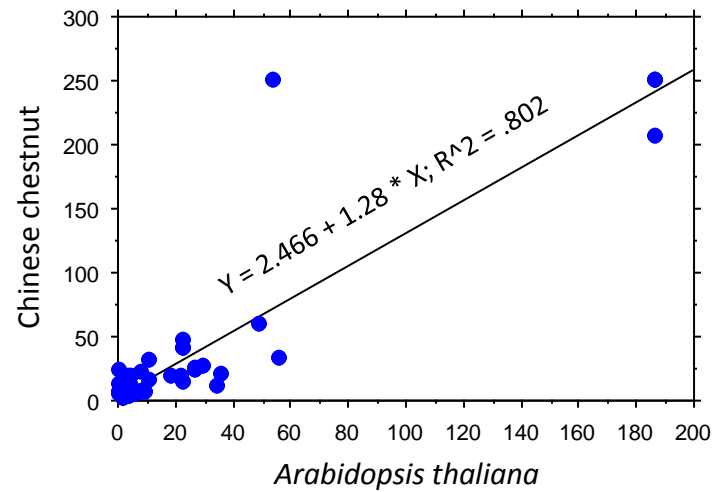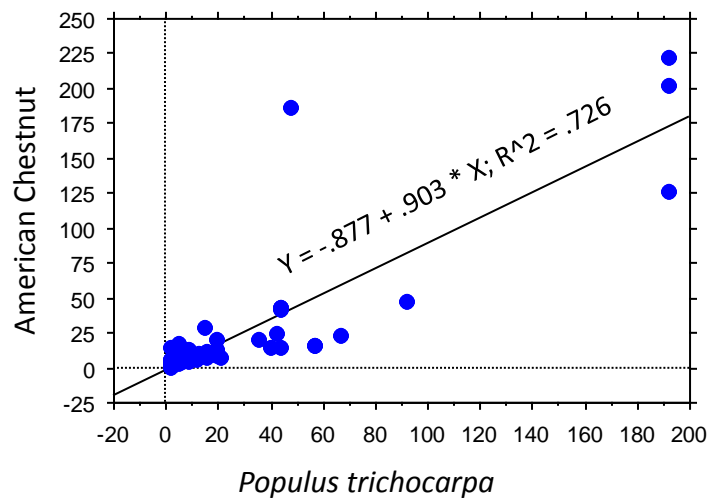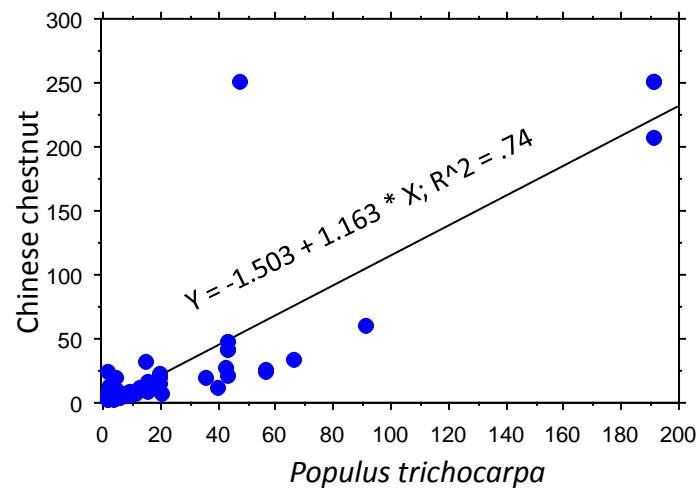

Supplement: Additional file 1 — Correlation between the number of genes per gene family in American chestnut, Chinese chestnut, Arabidopsis, and Populus. [file 1471-2229-12-38-S1.PDF]
